# Effect of Chewing Bicarbonate-containing Sugar-free Gum on the Salivary pH: An *in vivo* Study

**DOI:** 10.5005/jp-journals-10005-1330

**Published:** 2016-04-22

**Authors:** Raksha K Ballal, Sham S Bhat, Shenoy Shailesh Ramdas, Shrinidhi Ballal

**Affiliations:** 1 Senior Lecturer, Department of Pedodontics and Preventive Dentistry Yenepoya Dental College and Hospital, Mangaluru, Karnataka India; 2 Head, Department of Pedodontics and Preventive Dentistry Yenepoya Dental College and Hospital, Mangaluru, Karnataka India; 3 Senior Lecturer, Department of Pedodontics and Preventive Dentistry Yenepoya Dental College and Hospital, Mangaluru, Karnataka India; 4 Ex-Student, Department of Pedodontics and Preventive Dentistry Yenepoya Dental College and Hospital, Mangaluru, Karnataka India

**Keywords:** Gum chewing, Oral health, Salivary flow rate, Salivary pH, Sugar-free gum.

## Abstract

The objective of the study was to evaluate the effect of chewing gum on the salivary pH and to compare the effect of chewing bicarbonate-containing sugar-free gum on salivary pH against that of standard sugar-free gum. The experiment was carried out on 30 volunteers aged 20-22 years (mean age = 21 years) who fulfilled the inclusion criteria. The test gum was sugar-free greenmint-flavored bicarbonate-containing gum and the standard control was sugar-free spearmint-flavored gum. The pH was measured immediately using pH strips.

According to statistical analysis, the mean salivary pH of the bicarbonate gum at 0, 5, 10, 15 and 20 minutes is 6.9713, 6.5667, 6.4267, 6.3867 and 6.3233 respectively. There is decrease in pH from 0 to 20 minutes. According to Bonferroni, there was no significant difference in pH from 0 to 20 minutes, 10 to 20 minutes and 15 to 20 minutes, but there was a significant difference in salivary pH from 5 to 20 minutes (p = 0.014).

The mean salivary pH of the standard gum at 0, 5, 10, 15 and 20 minutes is 6.8767, 6.6067, 6.4200, 6.4027 and 6.3000 respectively. There is decrease in pH from 0 to 20 minutes. According to Bonferroni, there was no significant difference in pH from 0 to 20 minutes, 5 to 20 minutes, 10 to 20 minutes and 15 to 20 minutes. Thus, the higher salivary pH achieved with chewing bicarbonate gum compared with a standard sugar-free gum may have important oral health implications.

**How to cite this article:** Ballal RK, Bhat SS, Ramdas SS, Ballal S. Effect of Chewing Bicarbonate-containing Sugar-free Gum on the Salivary pH: An *in vivo* Study. Int J Clin Pediatr Dent 2016;9(1):35-38.

## INTRODUCTION

The oral health benefits of gum chewing are well-known.^1^ Chewing gum typically consists of sweetener, gum base flavoring agent and aromatic agent. Historically, chewing gum was sweetened with sucrose which contributed to tooth decay. Today more than 50% of chewing gums are sweetened with sugar contributes, such as polyol sweetener, artificial sweetener or both. Few studies have shown that oral bacteria do not use sugar substitutes to produce acids that demineralize the enamel or dentin.^2^

Chewing gum is a potent stimulator of salivary flow which increases buffering capacity and enhances clearance of food debris and microorganisms from the oral cavity. It also increases salivary flow, pH and plaque pH and can provide a vehicle for delivering medicaments, such as chlorhexidine, enzymes, fluoride and whitening agents.^3^

## REVIEW OF LITERATURE

Chewing gum stimulation of salivary flow (at the time of the pH minimum following exposure of plaque to carbohydrate) has been shown to cause a rapid increase in plaque pH (Jensen, 1986 a, b; Igarashi et al, 1988; Jensen and Wefel, 1989). Saliva is normally present in the mouth as a film of estimated thickness of 0.1 mm (Collins and Dawes, 1987) which, under unstimulated conditions, moves rather slowly over the surface of plaque (0.8-8 mm/minute; Dawes et al, 1989). The low velocity of the salivary film will allow metabolically produced acids from dental plaque to accumulate in the overlying saliva, which will retard the rate of acid clearance from plaque.

Chewing gum can provide a vehicle for delivering medicaments, such as chlorhexidine, enzymes, fluoride and whitening agents. Today, owing to its vast consumption worldwide, especially by youngsters, it has grown to be a multibillion-dollar industry and is now being largely used in combination with many other ingredients to benefit oral health, one such combination being chewing gum with sodium bicarbonate (baking soda). Chewing sodium bicarbonate gum for 10 minutes was found to raise the interproximal plaque pH from 4.3 ± 0.3 (n = 4) following an acidogenic challenge (toffee chewing) to a pH of 6.1 ± 0.61. Chewing bicarbonate gum would, thus, be expected to increase salivary pH as bicarbonate ions leach out from the gum.^4^

## AIMS AND OBEJCTIVES

- To evaluate the effect of chewing gum on salivary pH
- To compare the effect of chewing bicarbonate-containing sugar-free gum on salivary pH against that of sugar-free gum.

## MATERIALS AND METHODS

The experiments were carried out on 30 volunteers (dental students) aged 20-22 years (mean age 21 years) who fulfilled the inclusion criteria, which included the following: the subjects had to be nonsmokers, without any significant oral, dental or systemic diseases; not taking any medication likely to interfere with salivation; not wearing any intraoral appliances and not having an allergy to gum ingredients. Informed consent was obtained prior to the start of the study, which had been approved by the Institutional Ethical Committee.

### Chewing Gums

Two types of gum purchased from retail outlets were used in this investigation. The test gum was sugarfree greenmint-flavored bicarbonate-containing gum (Happy Dent, white; Perfeti Van Welle India Pvt Ltd., Gurgaon, Haryana, India) (Table 1) and the standard control was sugar-free spearmint-flavored gum (Wrigley’s Orbit; Wrigley India Pvt Ltd., Gurgaon, Haryana, India). The pellets of each gum type were of similar mass, each being 1.1 gm (Table 2).

**Table 1:** **Table 1:** Happy Dent Happy Dent-Base

		*N*		*Mean*		*Std. deviation*		*Minimum*		*Maximum*	
Base		30		6.4800		0.30895		6.00		7.00	
0		30		6.9713		0.32007		6.40		7.60	
5		30		6.5667		0.29283		6.20		7.20	
10		30		6.4267		0.27156		6.00		7.00	
15		30		6.3867		0.22854		5.80		7.00	
20		30		6.3233		0.24870		5.50		6.80	

F = 20.803; p < 0.001; vHS

**Table:** Multiple Comparisons Dependent variable: Happy Dent Base Bonferroni

*Time (I)*		*Time (J)*		*Mean difference (I-J)*		*P*	
Base		0		–0.4913		<0.001 VHS	
		5		–0.0867		1.000	
		10		0.0533		1.000	
		15		0.0933		1.000	
		20		0.1567		0.477	
0		5		0.4047		< 0.001 vHS	
		10		0.5447		< 0.001 vHS	
		15		0.5847		< 0.001 vHS	
		20		0.6480		< 0.001 vHS	
5		10		0.1400		0.820	
		15		0.1800		0.207	
		20		0.2433		0.014 S	
10		15		0.0400		1.000	
		20		0.1033		1.000	
15		20		0.0633		1.000	

VHS: Very highly significant; S: Significant

**Table 2:** **Table 2:** Orbit Orbit Base

		*N*		*Mean*		*Std. deviation*		*Minimum*		*Maximum*	
Base		30		6.4833		0.38245		5.50		7.20	
0		30		6.8767		0.28246		6.30		7.20	
5		30		6.6067		0.23771		6.00		7.00	
10		30		6.4200		0.21238		6.00		6.80	
15		30		6.4067		0.25452		6.00		7.20	
20		30		6.3000		0.20172		5.80		6.80	

F = 17.221; p < 0.001

**Table:** Multiple Comparisons Dependent variable: Orbit Base Bonferroni

*Time (I)*		*Time (J)*		*Mean difference (I-J)*		*P*	
Base		0		–0.3933		<0.001 VHS	
		5		–0.1233		1.000	
		10		0.0633		1.000	
		15		0.0767		1.000	
		20		0.1833		0.135	
0		5		0.2700		0.002 HS	
		10		0.4567		<0.001 vHS	
		15		0.4700		<0.001 vHS	
		20		0.5767		<0.001 vHS	
5		10		0.1867		0.117	
		15		0.2000		0.067	
		20		0.3067		<0.001 vHS	
10		15		0.0133		1.000	
		20		0.1200		1.000	
15		20		0.1067		1.000	

VHS: Very highly significant; HS: Highly significant

## EXPERIMENTAL PROCEDURE

The pellets of two types of gum were placed separately. The baseline pH was checked before the experiment was started. The participants were given a single pellet of Happy Dent white and were allowed to chew at their own pace for 2 minutes. The saliva was collected in a container and the pH was measured immediately using pH strips (BBR chemocraft) in order to minimize any time-based pH changes, e.g., loss of CO_2_ from the sample. And the color changes in the pH strips were compared with the BBR chemocraft color chart. The values were measured at 0, 5, 10, 15, and 20 minutes. The entire experiment was repeated with the second gum sample. The baseline pH was checked before repeating the experiment with the second gum sample. The interval between the two experiments was 20 minutes. This interval had been determined from a similar study after a series of pilot experiments and was found sufficient to allow pH to return to basal levels.^3^ The two experiments were carried out in a single session. The results were tabulated and analyzed using paired t-test and repeated measures analysis of variance (ANOVA).

## OBSERVATION AND RESULTS

**Graph 1:**
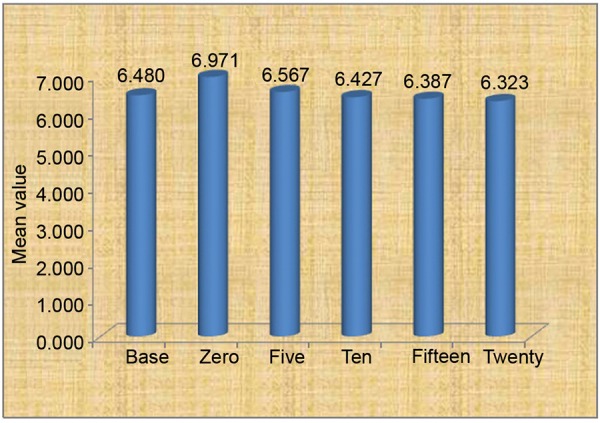
Mean value of Happy Dent

**Graph 2:**
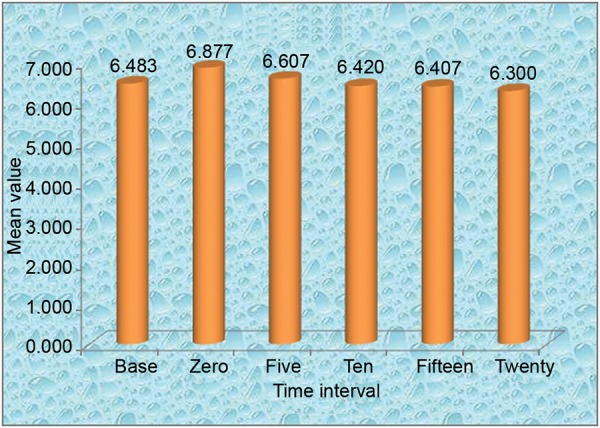
Mean value of Orbit

The results for the stimulated salivary pH using bicarbonate-containing sugar-free chewing gum and standard chewing gum are shown in Graphs 1 and 2 respectively. The mean salivary pH of the bicarbonate gum at 0, 5, 10, 15 and 20 minutes is 6.9713, 6.5667, 6.4267, 6.3867 and 6.3233 respectively. There is decrease in pH from 0 to 20 minutes. According to Bonferroni, there was no significant difference in pH from 0 to 20 minutes, 10 to 20 minutes and 15 to 20 minutes, but there was a significant difference in salivary pH from 5 to 20 minutes (p = 0.014).

The mean salivary pH of the standard gum at 0, 5, 10, 15 and 20 minutes is 6.8767, 6.6067, 6.4200, 6.4027, 6.3000 respectively. There is decrease in pH from 0 to 20 minutes. According to Bonferroni, there was no significant difference in pH from 0 to 20 minutes, 5 to 20 minutes, 10 to 20 minutes and 15 to 20 minutes. The average salivary pH obtained with bicarbonate gum at 0 minutes was greater than salivary pH of standard gum. At 5 minutes the average salivary pH of bicarbonate gum was less than that of standard gum, at 10 minutes it was greater than that of standard gum, at 15 minutes it was lesser than that of standard gum and at 20 minutes it was greater than that of standard gum.

## DISCUSSION

Chewing sugar-free gum is a convenient way to increase salivary flow and is promoted as an oral health aid. It also raises salivary and plaque pH and promotes enamel remineralization.^5^ It can also be used as a vehicle for delivering substances, such as chlorhexidine, enzymes, fluoride or bicarbonate ions. The protective effects of saliva are due to the presence of a variety of antimicrobial substances, growth factors and inorganic ions, such as calcium, phosphate and bicarbonate.

It has been shown that on chewing flavored gum, the salivary flow rate is initially increased but declines as the flavoring is lost from the gum^6^ and as the gum softens with chewing.^7^ This study was conducted to evaluate the effect of chewing gum on the salivary pH and also to compare the effect of chewing bicarbonate-containing sugar-free gum on the salivary pH against that of sugar-free gum.

The saliva sample was collected after chewing bicarbonate-containing sugar-free gum and the standard gum and then the pH was measured using pH strips. The pH meter requires greater quantity of saliva to provide the pH value. Due to difficulty in obtaining 3-5 ml of saliva at every 5-minute interval, the pH meter was not used for the study. The experiment showed that there was a statistically significant raise in the peak pH of bicarbonate-stimulated saliva than that produced by chewing the standard gum at 5-20 minutes interval.

In a similar study the pH of bicarbonate gum-stimulated saliva was higher than the pH of the saliva obtained by chewing the standard gum sample. Also the mean salivary flow rates for bicarbonate gum and control gum were greater than the unstimulated flow rate for all time points. However, the differences were significant only up to 15 minutes. There was no significant difference between the salivary flows evoked by the two types of gum at any of the time points.^3^

It is likely that raise in salivary pH was linked to an increase in salivary bicarbonate concentration. The bicarbonate gum pellets contain 4% (w/w) sodium bicarbonate and it is most likely that additional increase in salivary pH with the bicarbonate gum was due to bicarbonate ions leaching out from the gum.^3^ As this reservoir diminishes with time, the difference in pH of the saliva stimulated by each gum will decrease. A study by Rosenhek, Macpherson and Dawes suggested that most of the gum ingredients (sucrose) were lost after 10-15 minutes depending on the size of the sample. These data are consistent with the time course of salivary pH changes reduced by chewing bicarbonate gum in the present experiments.

According to a study, it was suggested that the buffering capacity of unstimulated saliva is low and it contains only a low concentration of bicarbonate (Dawes 1969; Jenkins, 1978). However, when the flow rate is increased the bicarbonate concentration in secretions from the major salivary glands is greatly increased (Dawes 1969, 1974), and bicarbonate is then by far the major buffer in saliva (Leung, 1951; Lilienthal, 1955).

During chewing gum stimulation of salivary flow there will be an increase in salivary film velocity associated with the increased flow rate (Dawes et al, 1989), better mixing of saliva in the mouth and also an increase in salivary buffering capacity. These factors may all, in part, be responsible for the elevation of the pH by the chewing gum.

Clinical studies involving the chewing of xylitol-containing gum daily for 2 years would appear to prove beneficial, since the caries increment of children in the gum-chewing groups was lower than that of control groups on a normal caries preventive program (Isokangar et al, 1988; Kandelman and Gagnon, 1990). Thus the higher salivary pH achieved with chewing bicarbonate gum, compared with the standard sugar-free gum, may have important oral health implications.^3^

## CONCLUSION

Gum chewing increases salivary flow rate and salivary pH and is also known to increase the plaque pH. The sugar-free chewing gum is said to have anticariogenic effect. Also, chewing bicarbonate gum increases the salivary pH as bicarbonate ions leach out from the gum. Therefore, a combination of the two, i.e., chewing a bicarbonate sugar-free gum, has additive effects to the oral health, and thus proves to be beneficial. It may, thus, be advisable for use under the dentist’s recommendation.
